# Extreme caution on the use of sirolimus for the congenital hyperinsulinism in infancy patient

**DOI:** 10.1186/s13023-017-0621-5

**Published:** 2017-04-14

**Authors:** Indraneel Banerjee, Diva De Leon, Mark J. Dunne

**Affiliations:** 1 Paediatric Endocrinology, Central Manchester University Hospitals NHS Foundation Trust (CMFT) and The University of Manchester, Oxford Road, Manchester, M13 9PT UK; 2 Congenital Hyperinsulinism Center, Division of Pediatric Endocrinology and Diabetes, The Children’s Hospital of Philadelphia, Philadelphia, PA 19104 USA; 3 Faculty of Biology, Medicine & Health, The University of Manchester, Oxford Road, Manchester, M13 9PT UK

**Keywords:** Sirolimus, Congenital hyperinsulinism in Infancy patient, mTOR, islet, hypoglycaemia

## Abstract

We have recently published on the limited effectiveness of sirolimus as a treatment option for hypoglycaemia as a consequence of hyperinsulinism. Our data oppose the view that mTOR inhibitors provide new opportunities for the treatment of patients with hyperinsulinism. We are not convinced by the argument that any benefit for some patients outweighs the potential and later long-term problems that accompany mTOR inhibition in the neonate. We also express the opinion that caution must be taken when repurposing/repositioning therapies in the field of rare disease.

Inappropriate insulin release from islet β-cells is the principal cause of sustained hypoglycaemia in the newborn and neonatal periods. Despite being first characterized more than 60 years ago, Congenital Hyperinsulinism in Infancy (CHI) still carries a significant risk of brain damage and more than 40% of affected children develop developmental delays and learning disabilities. Concurrent with advances in genetic diagnosis and nuclear medicine imaging has come significant progress in predicting the value of early surgical treatment of disease, which is now curative for some groups of patients. This is in stark contrast to progress in the area of medical treatment, which has seen little meaningful change for patients over the past 30 years [1]. Despite the fact that diazoxide and somatostatin receptor agonists are used off-label and carry significant side effects to patient well-being, they endure as mainline treatments because there are no alternatives. Unfortunately in the drug-unresponsive patient, surgery to remove up to 95% of the pancreas is still the most advantageous option for most patients in specialized treatment Centres; but this too carries significant short- and long-term complications, including iatrogenic diabetes. Whilst pilot clinical trials with novel compounds do offer some future long-term hope for new therapeutic options (soluble-glucagon, antagonists of the GLP-1 receptor and allosteric antibodies to the insulin receptor), this does not mitigate our current, daily dilemma in optimizing individualized treatment strategies towards either the surgical or medical management option, or both.

In 2014 the New England Journal of Medicine published on the successful use of the mTOR inhibitor Sirolimus in CHI patients who were unresponsive to diazoxide and Octreotide [2]. Despite early concerns about the use of this drug in the neonatal population [3], several case studies have subsequently appeared in the literature reporting the success of Sirolimus therapy in CHI with no reports of adverse outcomes [4–7]. The original paper by Senniappan and colleagues (2014) is not without weakness. It was based on just four patients and the proposed mechanisms of action used to justify the study were formulated on two pathological samples of tissues in which the genetic cause of CHI was confirmed in only one patient [8]. In a follow-up paper, the original authors then used gene expression profiles to seed an informatics-based study to reaffirm a role for mTOR inhibitors in suppressing β-cell expansion and proliferation [9]. However, this dataset was derived from pathological samples in which the CHI [neonatal] tissue was compared to the adult pancreas; which not surprisingly highlighted enrichment pathways relative to growth and therefore tissue expansion.

To address concerns about the widening use of Sirolimus in these difficult to treat patients, the experiences of two international specialist treatment Centres for CHI have been published. Szymanowski et al. (2016) report in a cohort of 10 patients, that the effectiveness of Sirolimus is far worse than that of either diazoxide or Octreotide, and that Sirolimus carries a greater risk of side-effects and short-term complications than either of the standard medications for CHI [10]. There was no evidence to support the proposed mechanisms of action of Sirolimus on β-cells. The expression of the *mTOR* gene was no different in control and CHI tissue (Fig. 1), and moreover the mTOR pathway is not implicated in the network of pathways causally-linked to disease. The authors of Szymanowski et al. (2016) also noted that one patient treated in the cohort stopped sirolimus after one year as drug efficacy was lost.

**Fig. 1:**
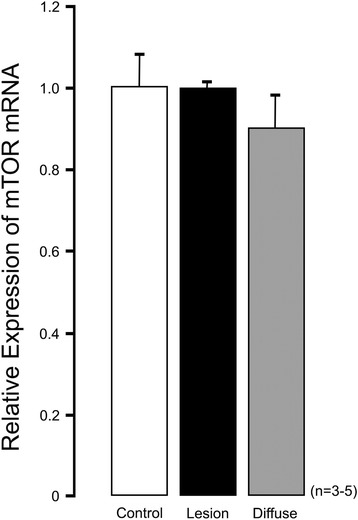
Relative expression of mTOR in CHI tissue. The relative expression of mTOR mRNA is no different in focal (*n* = 5 cases, ‘Lesion’) or diffuse CHI (*n* = 3 cases, ‘Diffuse’) when compared to age-matched controls (*n* = 4 cases)

The repositioning of drugs for the treatment of rare and orphan conditions is currently becoming increasing important and of strategic significance in global health alliances. As we seek to meet the challenges of replacing poorly-tolerated and unsatisfactory medications with repurposed/new, safer and more effective medications, it is important that this is carried out under stringent trial/pilot trial conditions. There should be strict regulation around the application of drugs in trial conditions and a robust process should be in place for the reporting of treatment failures and side-effects. In the field of rare diseases, the requirement to report negative data is arguably more important as the demands for new and/or more effective medications can be even more pressing.

## Conclusions

Sirolimus therapy for CHI has positive outcomes for some patients. However, we do not know who will benefit and we are not convinced by the argument that any benefit for some patients outweighs the potential and later long-term problems that accompany mTOR inhibition in the neonate. The absence of short-time side effects in reports claiming therapeutic success does not exclude long-term consequences from prolonged exposure, including the risk of malignancy [11]. In our experience, short-term effects from sirolimus were too detrimental to encourage long-term use.

I Banerjee (Manchester); D De Leon (Philadelphia); MJ Dunne (Manchester).

## Abbreviation

CHI: Congenital Hyperinsulinism in Infancy
